# Visual Outcomes of a New Hydrophobic Trifocal Intraocular Lens in Cataract Treatment: A Prospective Clinical Study

**DOI:** 10.1155/joph/2662730

**Published:** 2025-07-24

**Authors:** Andrea Janeková, Peter Mojžiš, Iveta Němcová, Marek Kačerík, Pavol Veselý, Lucia Hrčková

**Affiliations:** ^1^Eye Center Prague, Prague, Czech Republic; ^2^Faculty of Medicine in Hradec Kralove, Charles University, Hradec Kralove, Czech Republic; ^3^Department of Ophthalmology, Havlickuv Brod Hospital, Havlickuv Brod, Czech Republic; ^4^Department of Ophthalmology, Military University Hospital, Prague, Czech Republic; ^5^First Faculty of Medicine Charles University, Prague, Czech Republic; ^6^Eye Clinic Vidissimo, Trenčín, Slovakia; ^7^VESELY Eye Clinic, Bratislava, Slovakia

**Keywords:** aberration-neutral, diffractive–refractive, multifocal, presbyopia, single-piece

## Abstract

**Purpose:** To report the visual outcomes following bilateral implantation of a new trifocal intraocular lens (IOL) in patients with age-related cataracts.

**Methods:** This prospective, noncomparative, multicenter study assessed 126 patients undergoing cataract extraction followed by AT ELANA 841P IOL implantation. At 4–6 months postoperatively, refractive error and predictability, monocular uncorrected distance visual acuity (UDVA), corrected distance visual acuity (CDVA), uncorrected intermediate visual acuity (UIVA), distance-corrected intermediate visual acuity (DCIVA), uncorrected near visual acuity (UNVA), distance-corrected near visual acuity (DCNVA), and binocular contrast sensitivity were measured. The binocular defocus curve was evaluated at 3 months postoperatively.

**Results:** Postoperatively, most of the eyes (84.9%) were within ±0.5 D of spherical equivalent (SE) refraction and almost all eyes (98.9%) within ±1.0 D, with a mean SE value of −0.11 ± 0.37 D. Mean monocular CDVA was −0.06 ± 0.08 logMAR and UDVA was −0.01 ± 0.10 logMAR. Mean monocular uncorrected (UIVA and UNVA) and distance-corrected visual acuities (DCIVA and DCNVA) were 0.1 logMAR or better at intermediate (80 cm) and near (40 cm) distances. In the mean defocus curve, a continuous range of 0.1 logMAR or better vision from distance to near was observed. Percentages of eyes achieving CDVA, DCIVA, and DCNVA of 0.1 logMAR or better were 97.2%, 59.1% and 59.1%, respectively. Uncorrected visual acuity of 0.1 logMAR or better was achieved in 88.9% of the eyes at far, 57.1% at intermediate, and 44.4% at near distances. Contrast sensitivity was in the normal range of a phakic population at all spatial frequencies in all light conditions tested, photopic with glare and mesopic with and without glare.

**Conclusion:** Implantation of the new AT ELANA 841P IOL following cataract extraction is safe and effective. Visual acuities at all distances, refractive outcomes, and contrast sensitivity were favorable at 4–6 months postoperatively, providing patients with satisfactory far, intermediate, and near vision.

**Trial registration:** ClinicalTrials.gov identifier: NCT06247683

## 1. Introduction

Multifocal intraocular lenses (IOLs) are increasingly used in the management of presbyopia and cataract. To ensure surgeons can select the best option for their patients, a new hydrophobic trifocal IOL has recently been launched onto the market. The AT ELANA 841P features an aberration-neutral refractive–diffractive trifocal optic developed on the well-established CT LUCIA platform.

A bench study previously reported the detailed characteristics of the haptics and optic–haptic junction of the CT LUCIA 621PY IOL [1]. When compared to four other commercially available one-piece hydrophobic acrylic monofocal IOLs, the CT LUCIA 621PY consistently showed the greatest dimensions for both the haptics (length, thickness, surface area, and volume) and the optic–haptic junction (surface area and volume). In addition, the CT LUCIA 621PY showed the greatest angle of contact and highest capsular bag contact with the smallest haptic–capsular bag relationships change when subjected to compression [1]. Altogether, these features might be advantageous for the IOL positional stability in the capsular bag. These results were confirmed in an independent study using cadaver eyes [2]. Moreover, several clinical studies confirmed these predictions [3–5]. The authors reported excellent refractive outcomes, with a minimum of 90% of eyes within SE ± 1.0 D and mean values within ±0.25 D. Linear regression analysis of attempted versus achieved spherical equivalent (SE) showed a coefficient of determination close to 1, confirming a very predictable refractive correction [3]. No case of postoperative clinically significant IOL tilt, decentration, or IOL displacement was found.

More recently, a second bench study was published by the same authors, this time reporting detailed characteristics of the AT ELANA 841P geometry and mechanical behavior [6]. They showed that, in an experimental setting using compression wells to evaluate the haptic–capsular bag relationships, AT ELANA 841P showed the largest length of contact and largest contact zones of all hydrophobic samples tested as well as good axial alignment behavior and no decentration. These observations suggest that postoperatively, the IOL might demonstrate good positional stability within the capsular bag leading to refraction stability. In addition, the optical function of the AT ELANA 841P was previously tested in preclinical settings that aimed at predicting postoperative performance [7]. The AT ELANA 841P experimentally demonstrated good simulated visual acuity (VA) at far, intermediate (100 cm), and near (40 cm) distances independently of spectral and spherical aberration conditions. The simulated defocus curve, derived from the area under the modulation transfer function (MTF), resulted in simulated VA of 0.20 logMAR or better throughout a range of +0.5 D to −3.0 D. At 0.0 D of defocus, the predicted VA was 0.00 logMAR. The IOL simulated intermediate VA at −1.25 D was 0.1 logMAR (equivalent to an optical distance of 80 cm) with a near VA of 0.05 logMAR at −2.5 D (40 cm).

Altogether, the preclinical data suggest that the design of AT ELANA 841P may be advantageous in maintaining a stable optical position throughout time despite forces exerted by the capsular bag and may provide predictable visual outcomes after cataract surgery. Thus, this study reports, for the first time, the clinical results of patients treated with AT ELANA 841P for age-related cataract: visual and refractive outcomes, depth of focus, and contrast sensitivity (CS) 4–6 months after bilateral IOL implantation.

## 2. Materials and Methods

### 2.1. Study Design and Patients

This prospective, noncomparative, multicenter clinical investigation aimed at evaluating the safety and efficacy of a new trifocal IOL in patients with bilateral age-related cataracts.

The study was conducted at 11 sites located in the Czech Republic, Germany and Slovakia. The trial was carried out in accordance with the ethical principles stated in the Helsinki Declaration and in compliance with Good Clinical Practice (ISO 14155). The clinical investigation complied with all local and national applicable governmental regulations concerning the conduct of clinical investigations on human subjects. The trial was approved by the relevant competent authority and ethics committee/institutional review board in each participating country and institution (NMB 31608/2022, Czech Republic; 2022-620-f-S, Germany; approval without a reference number, Slovakia). Patients received comprehensive information about the potential risks and benefits associated with the procedure and constraints related to their participation in a clinical investigation and gave written informed consent to participate in the study prior to enrollment.

Adult patients of any gender with clinically significant bilateral age-related cataract, and no other major ocular pathology, eligible for implantation of a posterior chamber trifocal IOL, were enrolled in the study. The subjects had to have a potential binocular postoperative corrected distance VA (CDVA) of 0.2 logMAR or better in both eyes with preoperative corneal astigmatism < 1.0 D and requiring an IOL power within the available range of the investigational IOL (0.0 to +34.0 D). Main exclusion criteria included any acute, chronic, or uncontrolled systemic or ocular disease likely to increase operative risk or confound the outcome of the study (e.g., uncontrolled diabetes), clinically significant corneal abnormalities, previous intraocular or corneal/refractive surgery, degenerative visual disorders, use of systemic and/or ocular medications that may affect vision, and ocular conditions that may predispose the patient to future complications as per investigator's medical judgment.

All subjects received the same treatment. AT ELANA 841P IOL was implanted in both eyes with 1–15 days between the two implantations between March 13^th^, 2023 and March 26^th^, 2024.

### 2.2. IOL

The AT ELANA 841P (Carl Zeiss Meditec AG) is a posterior chamber intraocular IOL preloaded in a disposable sterile injector (BLUESERT injector). The device is a foldable, trifocal, aberration-neutral, single-piece C-loop IOL made of hydrophobic acrylic material which incorporates a component that absorbs UV light and is coated with periodate-oxidized heparin that has no pharmacological, immunological, or metabolic action. The AT ELANA 841P relevant characteristics are presented in Table 1. The calculation of the IOL power for each patient eye was performed using the IOLMaster 700 (Carl Zeiss Meditec AG) (one site used the IOLMaster 500). The recommended A-constant could be found on the IOL Con website (https://www.iolcon.org) and the “Barrett TK Universal II” formula was the most used for IOL power calculation. Other formulas were allowed to be used if better visual outcomes for the patient were expected. In all cases, emmetropia was targeted (target refraction +0.00 D/plano).

### 2.3. Surgical Procedure

The subject's eyes were prepared for the standard small-incision phacoemulsification cataract extraction procedure. In brief, administration of anesthesia was used followed by standard self-sealing clear corneal incision, continuous curvilinear capsulorhexis, hydrodissection of the natural lens, and conventional phacoemulsification for lens removal. The recommended incision size was ≥ 2.2 mm. At the end of the surgery, any residual ophthalmic viscoelastic device was thoroughly removed from the posterior chamber by irrigation/aspiration, and side ports and main incision were sealed by hydration. Intracameral antibiotics at the end of surgery, postoperative treatment, and medication were given according to the routine procedure in each study site.

### 2.4. Preoperative and Postoperative Assessments

Preoperatively, all patients underwent comprehensive evaluation including full medical history, slit lamp and fundus examination, objective and subjective refraction, determination of the dominant eye, monocular and binocular uncorrected distance visual acuity (UDVA) and CDVA, biometry, pupil size, and intraocular pressure.

Postoperatively, subjects were examined at Day 1 and Week 1 following each eye surgery and then for both eyes at 1, 3, and 4–6 months (120–180 days after the surgery of the second eye). Clinical outcomes of interest at 4–6 months were monocular UDVA, CDVA, uncorrected intermediate VA [UIVA], distance-corrected intermediate VA [DCIVA], uncorrected near VA [UNVA], and distance-corrected near VA [DCNVA]) and subjective SE refraction and binocular distance CS in mesopic conditions with and without glare and in photopic conditions with glare. The distance-corrected binocular defocus curve was tested at 3 months in photopic conditions.

SE refraction was assessed at a defined distance of 4 m (±12 cm). To adjust for optical infinity SE, the sphere was converted using (sphere −0.25 D). Standard clinical practice was followed by the surgeon by using the Clinical Trial Suite (CTS) (M&S Technologies, Inc., Niles, IL, USA).

Monocular corrected and uncorrected VAs were measured on an automated ETDRS chart using the CTS that provided descending logMAR charts with 100% contrast proportionally spaced SLOAN letters at the following distances: far 400 ± 12 cm, intermediate 80 ± 2 cm, and near 40 ± 1 cm. The luminance of the charts was standardized at 85 cd/m^2^. All VAs were expressed in logMAR units.

The same measurement conditions were used for measuring the binocular defocus curves. Testing was performed in photopic conditions. To obtain the defocus curve, VA was measured first with the distance best correction and then subsequently in 0.5 D defocus steps between −4.00 and +1.50 D using trial lenses. The results were recorded in logMAR.

Binocular far CS was measured with distance best correction using the Optec 6500 Vision Tester or Functional Vision Analyzer (Stereo Optical, Chicago, IL, USA). CS was measured at 1.5, 3, 6, 12, and 18 cpd spatial frequencies, under mesopic (3 cd/cm^2^), mesopic with glare, and photopic with glare (85 cd/cm^2^) conditions. Before starting CS measurements, the patient's pupil sizes under photopic and mesopic conditions were tested for both eyes (infrared Pupillometer VIP-300, NeurOptics, Inc., Irvine, CA, USA). After adaptation to the low-light condition (at least 10 min), mesopic testing was carried out first. Testing was performed twice for each patient at each test condition. The results of the CS measurement were recorded in logCS units.

Nondirected questioning was conducted to inquire about the patients' binocular visual symptoms, such as halo, glare, starburst, distortion, double images, and other phenomena. Visual symptoms were categorized and graded as mild, moderate, or severe. Clinically significant symptoms were reported as adverse events (AEs).

### 2.5. Statistical Analysis and Sample Size

Statistical analysis was performed using SAS Version 9.4 (SAS Institute Inc., Cary, USA). Data were summarized using descriptive statistics for all herein presented parameters: Categorical variables were presented as numbers and percentages of subjects per category, whereas continuous variables were presented using mean ± standard deviation (SD) and ranges. In all cases, a *p* value less than 0.05 was considered statistically significant.

Sample size calculation was conducted using the software PASS 2022 Version 22.0.2 and was based on the hypothesis that the CDVA at 4–6 months is ≤ 0.02 logMAR. Assuming a dropout rate of 5%, 135 subjects were enrolled in this study to result in 126 evaluable subjects for the full analysis set. This sample size was considered sufficient with a desired power of 80% for testing superiority of an expected mean CDVA of −0.01 logMAR (using a one-sided one-sample *t*-test with a Type-I error rate of 0.025, equivalent to a two-side test with a Type-I error rate of 0.05, and SD of 0.12 logMAR). The expected mean CDVA of −0.01 logMAR was an assumption made based on CT LUCIA 611P clinical trial results.

## 3. Results

### 3.1. Patient Demographics and Preoperative Measurements

This prospective clinical trial analyzed 252 eyes from 126 patients, of which, 72 were female (57.1%). One patient (1/127) discontinued the study after IOL implantation for health reasons unrelated to cataract surgery. The mean patient age was 63.3 ± 8.9 years (ranging from 33 to 81 years). As per the LOCS III grading scale [8], NO1/NO2 cataract nuclear opacity was found in 73.4% of the eyes and C1/C2 cortical opacity in 76.6% of the eyes. All eyes mean preoperative monocular CDVA was 0.15 ± 0.20 logMAR. All eyes were implanted with the AT ELANA 841P IOL, with a mean spherical IOL power of 22.02 ± 2.85 D (ranging from 14.00 to 31.50 D).  Table 2 summarizes the patient's preoperative demographics and measurements.

### 3.2. Refractive Outcomes

**Figure 1:**
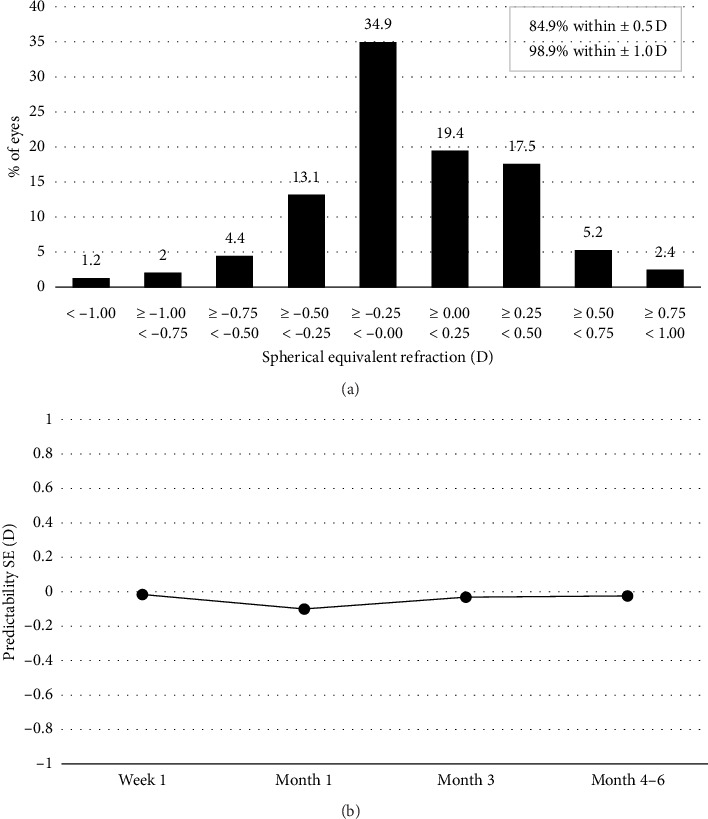
Distribution of spherical equivalent refraction at 4–6 months postoperatively (a) and refraction predictability over time (b).

At 4–6 months postsurgery, mean subjective SE refraction was −0.11 ± 0.37 D (ranging from −1.50 to 0.75 D).  Figure 1(a) shows the postoperative distribution of SE refraction, with most eyes (84.9%) being within ±0.5 D, and almost all eyes (98.9%) within ±1.0 D. More than half of the eyes (54.3%) were within ±0.25 D.

Predictability was calculated by subtracting SE (expected) from SE (postop), SE (expected) being the predicted residual refraction calculated by the IOLMaster based on the implanted IOL power and SE (postop) being the infinity adjusted SE at the respective postoperative visit.  Figure 1(b) shows the IOL refraction predictability that takes into account the lens A-constant. From 3 months onwards, refraction predictability was stable and close to 0, showing good accuracy between predicted and achieved refraction.

### 3.3. VA Outcomes

**Figure 2:**
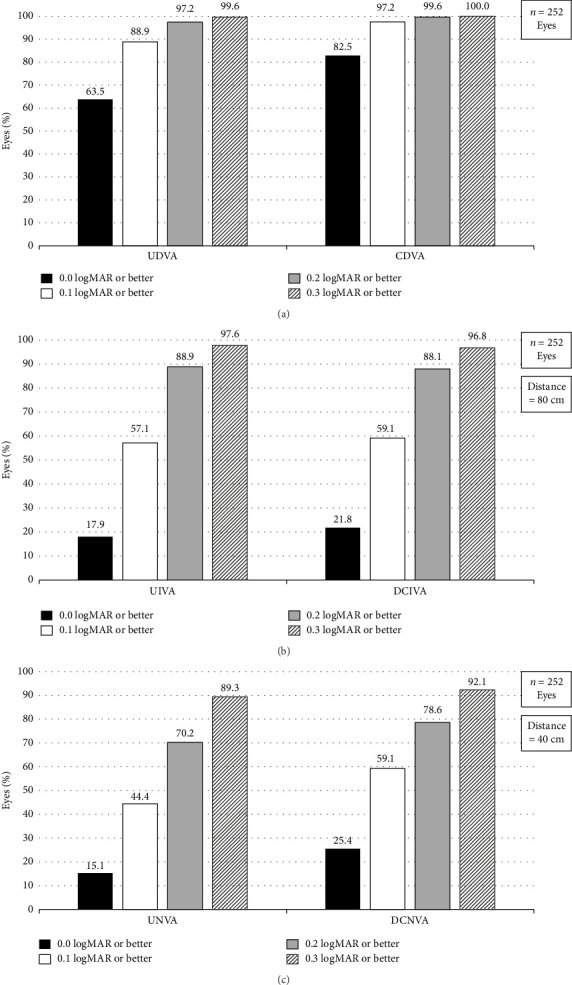
Distribution of monocular uncorrected distance visual acuity (UDVA) and corrected distance visual acuity (CDVA) in panel (a), monocular uncorrected intermediate visual acuity (UIVA) and distance-corrected intermediate visual acuity (DCIVA) in panel (b), and monocular uncorrected near visual acuity (UNVA) and distance-corrected near visual acuity (DCNVA) in panel (c) at 4–6 months postoperatively.

At 4–6 months following the AT ELANA 841P IOL implantation, the mean monocular CDVA was −0.06 ± 0.08 logMAR (ranging from −0.28 to 0.22) in all implanted eyes (*n* = 252). Mean monocular CDVA change from baseline (CFB) was −0.21 ± 0.21 logMAR. Mean monocular UDVA was −0.01 ± 0.10 logMAR (ranging from −0.26 to 0.42) in all implanted eyes and a gain of 4 lines with a mean CFB of −0.46 ± 0.29 logMAR providing patients with excellent distance VA (values presented in Table 3). At the postoperative visit 4–6 months, 82.5%, 97.2%, 99.6%, and 100% of the eyes had CDVA of 0.0 logMAR or better, 0.1 logMAR or better, 0.2 logMAR or better, and 0.3 logMAR or better, respectively (Figure 2(a)). Similarly, 63.5%, 88.9%, 97.2%, and 99.6% of the eyes had UDVA of 0.0 logMAR or better, 0.1 logMAR or better, 0.2 logMAR or better, and 0.3 logMAR or better, respectively (Figure 2(a)).

At intermediate vision (80 cm), more than half of the eyes achieved UIVA of 0.1 logMAR or better (57.1%). Cumulatively, 88.9% and 97.6% of the eyes attained 0.2 logMAR or better and 0.3 logMAR or better, respectively. Results were very similar for DCIVA with 59.1%, 88.1%, and 96.8% of the eyes reaching 0.1, 0.2, and 0.3 logMAR or better, respectively (Figure 2(b)). Mean monocular UIVA and DCIVA were both measured at 0.10 ± 0.10 logMAR (range extending from −0.12 to 0.46 and from −0.16 to 0.44, respectively) (Table 3).

At near distance (40 cm), 59.1% and 44.4% of eyes reached DCNVA and UNVA of 0.1 logMAR or better, respectively. Cumulatively, DCNVA of 0.2 and 0.3 logMAR or better was found in 78.6% and 92.1% of the eyes, respectively. Similarly, 70.2% and 89.3% of the eyes achieved UNVA of 0.2 and 0.3 logMAR, respectively (Figure 2(c)). Mean monocular near VA was 0.10 ± 0.13 logMAR (ranging from −0.20 to 0.50 logMAR) for DCNVA and 0.14 ± 0.13 logMAR (ranging from −0.18 to 0.50 logMAR) for UNVA (Table 3).

### 3.4. Depth of Focus

**Figure 3:**
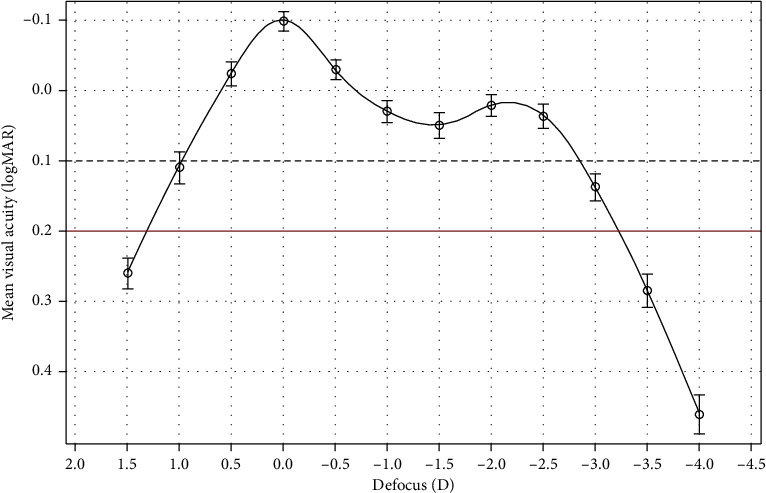
Mean binocular distance-corrected defocus curve at 3 months. Error bars represent the standard deviation, and penalized B-splines were used to smooth the defocus curve.

The defocus curve measured at 3 months postoperatively is shown in Figure 3. The mean binocular distance-corrected VA was better than 0.1 logMAR across the whole defocus range from +0.5 D (distance) to −2.5 D (near) (gray dashed line in Figure 3). A peak in mean VA for distance vision at 0.0 D was observed (−0.10 ± 0.08 logMAR) followed by a steady decrease to reach a mean VA of 0.03 ± 0.10 logMAR to 0.05 ± 0.10 logMAR at the intermediate distance (80 cm, Figure 3, between −1.0 and −1.5 D) and of 0.04 ± 0.10 logMAR at near distance (40 cm, Figure 3, −2.5 D).

In addition, the mean value of all eyes implanted with the AT ELANA 841P IOL met the ISO 11979-7:2024 defocus performance requirement for full visual range IOLs that implies distance-corrected VA at 1 m, 66, 50, and 40 cm to be 0.2 logMAR or better (represented by the red continuous line in Figure 3). Overall, the depth of focus from 0.0 D with VA of 0.2 logMAR or better spanned over 3.0 D.

### 3.5. CS

**Figure 4:**
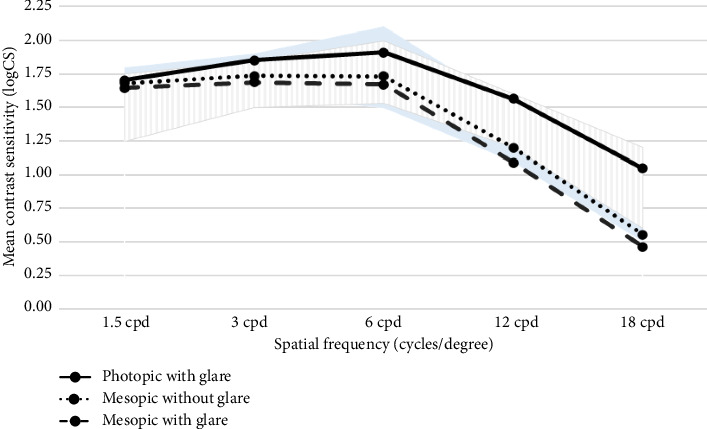
Mean binocular contrast sensitivity at 4–6 months in photopic with glare, mesopic without glare, and mesopic with glare light conditions. Background areas represent normal range distribution of subjects aged 40–49 years in daylight with glare (light blue) and daylight without glare (gray hatched) conditions [9].

Figure 4 shows the mean binocular CS tested at 4–6 months postoperatively. As expected, although mesopic CS (with and without glare) was lower than photopic CS, all three light conditions showed a similar performance profile with the highest contrast at 6 cpd. The addition of glare to the mesopic condition did not affect the results much. In addition, all three light condition profiles were encompassed within the normal range distribution of subjects aged 40–49 years in daylight conditions with glare and daylight conditions without glare [9] as represented by the background areas (light blue and gray hatched, respectively). This suggests that patients implanted with the AT ELANA 841P IOL were able to restore CS to the same level as the phakic middle-aged population exposed to daylight conditions.

### 3.6. Additional Outcomes

Finally, nondirected patient questioning at the 4–6 months visit did not reveal any types of severe halos or dysphotopsia. In all cases, eye-related AEs were resolved before the end of the study. No serious eye-related AEs were recorded.

## 4. Discussion

The results of this 4–6 months prospective study revealed that the bilateral implantation of the AT ELANA 841P IOL following cataract extraction was safe and effective. Patients restored high levels of VA and CS. The IOL demonstrated good refractive stability from 3 months onwards.

Preclinical laboratory studies had already set high postoperative expectations by reporting promising results regarding the optical function of the IOL [7]. In addition, the IOL-specific haptics design and geometry allow the IOL to maintain consistent contact with the capsular bag that indicates good intraocular positional stability and a predictable visual outcome after surgery. Indeed, the predecessor monofocal versions of the IOL (CT LUCIA series) that share the same haptic design have proven clinical records for good refractive predictability achieving emmetropia postoperatively [3–5].

Moreover, the AT ELANA 841P IOL function is based on a refractive/diffractive aspheric aberration-neutral optic design with 47.5% of light distributed to far focus, 17.5% to intermediate, and 35% to near. The IOL has a +1.66 D power addition to intermediate vision and a +3.33 D power addition to near vision. This study reports for the first time the visual outcome profile that one can expect upon AT ELANA 841P implantation following cataract surgery. These visual outcomes were comparable to similar IOLs that are on the market (particularly the AcrySof IQ PanOptix TFNT00, Alcon, and the TECNIS Synergy ZFR00V, J&J), with mean monocular UDVA ranging between 0.00 and 0.12 logMAR and mean monocular CDVA ranging from −0.01 to 0.05 logMAR when measured at 3–6 months postoperatively [10–16]. In this study, we reported the mean monocular CDVA of −0.06 ± 0.08 logMAR. Moreover, Rosen et al. [17] reported in a meta-analysis that examined published data on multifocal IOLs' visual performance in presbyopic patients with cataract or having refractive lens exchange, a mean postoperative monocular UDVA of 0.05 logMAR in 8797 eyes. Our study reported the mean monocular UDVA of −0.01 ± 0.10 logMAR with a high percentage of eyes reaching 0.1 logMAR or better (88.9%).

At intermediate distance, more than half of the eyes implanted with the AT ELANA 841P IOL reached 0.1 logMAR or better for both monocular UIVA (57.1%) and DCIVA (59.1%) with mean UIVA and mean DCIVA of 0.10 ± 0.10 logMAR equally. Our results were comparable with other studies that reported at 3–6 months postoperatively that, for the AcrySof IQ PanOptix and the TECNIS Synergy devices, mean monocular UIVA ranged from 0.1 to 0.3 logMAR and DCIVA ranged from 0.1 to 0.2 logMAR when measured at distances varying from 60 to 80 cm [11, 18, 19]. Moreover, Chang et al. [12] reported that monocularly 80.2% of the eyes implanted with Synergy reached DCIVA (66 cm) of 20/25 or better (∼0.1 logMAR) at 6 months, while Chang et al. [18] found that 63% of the eyes implanted with PanOptix reached 20/25 or better at 3 months.

At near vision, 44.4% (uncorrected) and 59.1% (distance-corrected) of the patients' eyes implanted with the AT ELANA 841P reached 0.1 logMAR or better and more than 70% reached 0.2 logMAR or better. Mean monocular UNVA was 0.14 ± 0.13 logMAR and mean DCNVA was 0.10 ± 0.13 logMAR. Data reported for the Synergy and PanOptix devices were similar. At 3–6 months postoperatively, mean monocular DCNVA measured at 40 cm ranged between 0.08 and 0.23 logMAR [11, 12, 15, 18]. Chang et al. [12] and Chang et al. [12, 18] reported that up to 70.2% and 85% of eyes implanted with the Synergy and PanOptix, respectively, reached monocular DCNVA (40 cm) of 20/25 or better.

The AT ELANA 841P specific IOL design profile has now clinically demonstrated good refractive performance with target refraction achieved with a slight myopic value (mean SE of −0.11 ± 0.37 D at 4–6 months postsurgery) and over 84% of eyes within ±0.5 D of SE refraction. From 3 months onwards, refraction was stable. To further understand the IOL behavior, it would be necessary to evaluate in further clinical studies, the stability of the IOL regarding tilt and decentration. In an unsystematic review of 30 articles on the topic, Chen et al. [20] concluded that a deep understanding of IOL tilt and decentration is of major importance for individual estimation of patient postoperative outcomes, and IOL design should be chosen carefully, especially if existing preconditions indicate uncertain postoperative position accuracy.

In this trial, it seems that patients were treated earlier than in other clinical studies at the stage of cataract with mild to moderate intensity. In fact, 73.4% of the eyes were found with NO1/NO2 nuclear opacity and almost the same portion of eyes (76.6%) had C1/C2 cortical opacity (according to the LOCS III grading scale) [8]. This is reflected in preoperative measurements with a relatively good mean monocular CDVA of 0.15 ± 0.20 logMAR. However, a mean gain of 2 lines in CDVA and 4 lines in UDVA was observed after implantation when compared to preoperative measurements suggesting a true benefit even for such a population.

Binocular defocus curves simulated from bench testing measurements of MTF indicated that the AT ELANA can deliver approximately 0.1 logMAR or better VA at far, intermediate, and near distances [7]. The results of this clinical study have now demonstrated that the AT ELANA 841P surpassed the optical bench results prediction. At 3 months postsurgery, binocular mean VA for distance vision at 0.0 D was excellent (−0.10 ± 0.08 logMAR). At intermediate distance between defocus points −1.0 and −1.5 D or 80 cm, mean VA reached between 0.03 ± 0.10 logMAR and 0.05 ± 0.10 logMAR and 0.04 ± 0.10 logMAR at near distance (defocus point −2.5 D or 40 cm). Thus, mean VA was better than 0.1 logMAR between +0.5 and −2.5 D and was at 0.2 logMAR or better in the interval of +1.0 and −3.0 D at 3 months postoperatively, offering a satisfactory long range of vision.

Previous published studies evaluated the performance of similar IOLs, the trifocal AcrySof PanOptix TFNT00 (Alcon), and the hybrid multifocal-extended-range-of-vision TECNIS Synergy ZFR00V (J&J), both IOLs designed to provide a good range of vision from distance to near.

Several authors reported that, at 3 months postoperatively, the mean binocular CDVA and DCNVA were significantly better for the Synergy IOL group than those for the PanOptix IOL group at distance and near (40 and 33 cm). The overall defocus range for 20/32 or better (∼0.2 logMAR or better) vision was approximately 0.4 D more with the Synergy IOL than with the PanOptix [21]. Moreover, the Synergy IOL defocus curve was approximately 0.5 lines above that of PanOptix IOL at 0.0, −1.5, and −2.5 D defocus and was approximately 1 line better than the PanOptix IOL at −3.0 D defocus, −3.5 D defocus, and −4.0 D defocus [21]. Nonetheless, both IOLs demonstrated a mean VA of 0.1 logMAR or better from +0.5 to −3.0 D. Regarding the AcrySof IQ PanOptix IOL, the near peak of the defocus curve was observed at a defocus of −2.00 and −2.50 D, which aligns with the recommended 40 cm near working distance, whereas a trend to obtain wider range of functional focus with the Synergy IOL was observed with mean VA of 0.2 logMAR or better to approximately −3.5 D (or 29 cm) [12, 18, 22–24]. Thus, the AT ELANA 841P depth of focus is comparable to other similar hydrophobic trifocal IOLs on the market. Although only a comparative clinical trial testing the three IOLs would confirm this literature outcome analysis, it is foreseen that the performance of the IOLs would show a slight difference in the intermediate vision. It has been reported that the PanOptix IOL has an intermediate focal point shift from 80 to 60 cm, and the intermediate performance of the IOL is slightly better at 60 cm compared to 80 cm [25, 26]. In contrast, even though the AT ELANA 841P was designed for best corrected intermediate vision at 80 cm, the IOL showed continuous good vision from 60 to 80 cm with mean binocular distance-corrected VA of 0.03 ± 0.10, 0.05 ± 0.10 and 0.02 ± 0.9 logMAR at defocus points −1.0, −1.5, and −2.0 D, respectively. Thus, the IOL also complies with the ISO 11979-7:2024 full visual range IOLs requirement for intermediate vision to be equal to or superior to 0.2 logMAR at 66 cm.

Although trifocal IOLs can restore vision across a long range of distances, they are generally associated with visual symptoms when compared to monofocal IOLs [27–30]. In this study, nondirected patient questioning at the 4–6 months visit did not reveal any type of severe halos or dysphotopsia. Further clinical studies over a longer period of time will be necessary to fully evaluate the kind and rate of dysphotopic disturbances the IOL might generate.

Moreover, CS attenuation is an inherent disadvantage of multifocal IOLs due to the light distribution to multiple focal points. Under both photopic and mesopic conditions, CS is significantly higher for monofocal IOLs than their multifocal counterparts [16]. However, the results of this study showed that patients bilaterally implanted with the AT ELANA 841P IOL showed very good levels of CS in all light conditions tested (photopic with glare and mesopic with and without glare) and all spatial frequencies. In fact, all three light condition profiles evaluated for eyes implanted with the AT ELANA 841P IOL were encompassed within the mean range of CS of healthy individuals (40–49 years) exposed to daylight condition with and without glare [9]. This suggests that the AT ELANA 841P IOL maintains levels of CS (photopic and mesopic, with and without glare) comparable to the levels observed in healthy phakic middle-aged individuals exposed to daylight with and without glare.

The main limitations of the study were as follows: the short-term follow-up (4–6 months) that does not capture longer-term complications such as posterior capsular opacification (PCO), IOL decentration or late visual disturbances, lack of a study control group, and absence of a validated patient-reported outcome (PRO) survey. In this study, nondirected patient questioning was conducted and did not reveal any severe type of dysphotopsia. However, subjective symptom reporting may underreport minor but clinically relevant visual phenomena. Validated questionnaires such as Catquest-9SF for assessing general PRO visual functioning and NEI VFQ-25 questionnaire including questions targeting visual phenomena such as halos, glare, starbursts, distortion, double images, and other related symptoms could provide a more comprehensive understanding of patient satisfaction and visual quality following cataract surgery.

Finally, to fully picture the abilities and limits of this new trifocal IOL on the market, further clinical data are expected regarding the IOL photic-phenomena profile, binocular visual performances, tilt and decentration behavior, development of PCO, and IOL-related AEs on a longer study follow-up time.

## 5. Conclusion

In conclusion, the results of this clinical study have demonstrated that following implantation of the new AT ELANA 841P IOL, cataract patients recovered very good vision. The IOL-specific optic and haptic designs allow good visual and refractive performance, offering a continuous range of vision of 0.1 logMAR or better from far to intermediate and near distances, and robust performance under various light conditions. Results were comparable to other trifocal hydrophobic single-piece C-loop IOLs available on the market.

## Figures and Tables

**Table 1:** Relevant characteristics of the new single-piece trifocal AT ELANA 841P IOL.

IOL characteristics	AT ELANA 841P
Material	Hydrophobic acrylic (Acrylmex)
Haptic design	Modified C-loop, step-vaulted, 0° angulation
Optic/overall diameter (mm)	6.0/13.0
Asphericity	Aberration-neutral
Optical design	Refractive–diffractive, trifocal (+1.66 D intermediate addition, +3.33 D near addition)
Anti-PCO edge	360° sharp edge on the back surface
Diopter range (D)	0.0 to +34.0 (0.5 increments)
Injector	Preloaded single-use BLUESERT
IOL coating	p-heparin^∗^

^∗^Fragment of heparin with no pharmacological, immunological, or metabolic action.

**Table 2:** Preoperative demographics of participants implanted with the AT ELANA 841P IOL shown as means, standard deviations (SD), and ranges.

Parameter	Values
Patients (*n*)	126
Eyes (*n*)	252
Gender, male/female (*n*)	54/72
Age (years)	63.3 ± 8.9 (33–81)
AL (mm)	23.47 ± 1.01 (21.25–26.08)
ACD (mm)	3.20 ± 0.35 (2.32–4.09)
Keratometry (D)	
Corneal astigmatism	0.50 ± 0.26 (0.00–1.16)
Corneal power (flat meridian)	43.12 ± 1.55 (38.63–47.23)
Corneal power (steep meridian)	43.62 ± 1.58 (39.30–47.97)
Subjective SE refraction (D)	0.21 ± 2.09 (−7.50–5.88)
Preoperative monocular visual acuity (logMAR)	
CDVA	0.15 ± 0.20 (−0.24–0.90)
UDVA	0.45 ± 0.29 (−0.08–1.54)
IOL power implanted sphere (D)	22.02 ± 2.85 (14.00–31.50)

*Note: n*, number of subjects (full analysis set).

Abbreviations: ACD, anterior chamber depth; AL, axial length; CDVA, corrected distance visual acuity; SE, spherical equivalent; UDVA, uncorrected distance visual acuity.

**Table 3:** Monocular corrected and uncorrected visual acuity at 4–6 months after implantation of the AT ELANA 841P shown as means, standard deviations (SD), and ranges.

	Visual acuity (logMAR)
CDVA	−0.06 ± 0.08 (−0.28–0.22)
CDVA (CFB)	−0.21 ± 0.21 (−1.00–0.12)
UDVA	−0.01 ± 0.10 (−0.26–0.42)
UDVA (CFB)	−0.46 ± 0.29 (−1.46–0.24)
DCIVA	0.10 ± 0.10 (−0.16–0.44)
UIVA	0.10 ± 0.10 (−0.12–0.46)
DCNVA	0.10 ± 0.13 (−0.20–0.50)
UNVA	0.14 ± 0.13 (−0.18–0.50)

Abbreviations: CDVA, corrected distance visual acuity; CFB, change form baseline; DCIVA, distance-corrected intermediate visual acuity; DCNVA, distance-corrected near visual acuity; UDVA, uncorrected distance visual acuity; UIVA, uncorrected intermediate visual acuity; UNVA, uncorrected near visual acuity.

## Data Availability

The data that support the findings of this study are available from the corresponding author upon reasonable request. The data are not publicly available due to privacy or ethical restrictions.

## References

[1] Borkenstein A. F., Borkenstein E.-M. (2022). Geometry of Acrylic, Hydrophobic IOLS and Changes in Haptic-Capsular Bag Relationship According to Compression and Different Well Diameters: A Bench Study Using Computed Tomography. *Ophthalmology and Therapy*.

[2] Zhang L., Schickhardt S., Auffarth G. U. (2022). An Experimental Laboratory Study Using the Miyake-Apple Posterior View Technique to Investigate the Dynamics Between Capsular Bags and Different IOL Models. *Journal of Refractive Surgery*.

[3] Schallhorn S. C., Teenan D., Venter J. A., Schallhorn J. M., Hannan S. J. (2023). Early Clinical Experience With a New Hydrophobic Acrylic Single-Piece Monofocal Intraocular Lens. *Clinical Ophthalmology*.

[4] Hernández-Martínez A., Díaz-del-Rio M. A., Ruiz-Santos M., Ruiz-Mesa R., Tañá-Rivero P. (2022). Refractive and Visual Outcomes of a Monofocal Non-Constant Aberration Aspheric Intraocular Lens. *Clinical Ophthalmology*.

[5] García-Tomás B., Marín-Sánchez J. M., García-Elskamp C., Alcon-Ruiz E., Montesinos-López L., García Martínez-Lozano B. (2023). Clinical Outcomes of a Monofocal, Optimized, Aspheric, Hydrophobic Acrylic Intraocular Lens Implant. *Clinical Ophthalmology*.

[6] Borkenstein A. F., Borkenstein E.-M. (2024). *Geometry of Modern Presbyopia-Correcting Intraocular Lenses and Changes in Haptic-Capsular Bag Behavior According to Compression and Different Well Diameters: A Bench Study Using Computed Tomography*.

[7] Łabuz G., Yan W., Khoramnia R., Auffarth G. U. (2023). Optical-Quality Analysis and Defocus-Curve Simulations of a Novel Hydrophobic Trifocal Intraocular Lens. *Clinical Ophthalmology*.

[8] Chylack L. T., Wolfe J. K., Singer D. M. (1993). The Lens Opacities Classification System III. The Longitudinal Study of Cataract Study Group. *Archives of Ophthalmology*.

[9] Hohberger B., Laemmer R., Adler W., Juenemann A. G. M., Horn F. K. (2007). Measuring Contrast Sensitivity in Normal Subjects With OPTEC 6500: Influence of Age and Glare. *Graefes Archive for Clinical and Experimental Ophthalmology*.

[10] Monaco G., Gari M., Di Censo F., Poscia A., Ruggi G., Scialdone A. (2017). Visual Performance After Bilateral Implantation of 2 New Presbyopia-Correcting Intraocular Lenses: Trifocal Versus Extended Range of Vision. *Journal of Cataract & Refractive Surgery*.

[11] Sahin V., Unal M., Ayaz Y. (2023). Outcomes After Bilateral Implantation of Acrysof IQ Panoptix Trifocal Intraocular Lens: A Prospective Interventional Study. *Medical Hypothesis, Discovery and Innovation Ophthalmology*.

[12] Chang D. H., Hu J. G., Lehmann R. P., Thompson V. M., Tsai L. H., Thomas E. K. (2023). Clinical Performance of a Hybrid Presbyopia-Correcting Intraocular Lens in Patients Undergoing Cataract Surgery in a Multicenter Trial. *Journal of Cataract & Refractive Surgery*.

[13] Jeon S., Moon K., Kwon H. (2023). Long-Term Clinical Outcomes After Trifocal Intraocular Lens Implantation: A Retrospective Observational Study. *Journal of Refractive Surgery*.

[14] Moshirfar M., Stapley S. R., Corbin W. M. (2022). Comparative Visual Outcome Analysis of a Diffractive Multifocal Intraocular Lens and a New Diffractive Multifocal Lens With Extended Depth of Focus. *Journal of Clinical Medicine*.

[15] Moshirfar M., Stoakes I. M., Theis J. S. (2023). Assessing Visual Outcomes: A Comparative Study of US-FDA Premarket Approval Data for Multifocal and EDOF Lens Implants in Cataract Surgery. *Journal of Clinical Medicine*.

[16] Imburgia A., Gaudenzi F., Mularoni K., Mussoni G., Mularoni A. (2022). Comparison of Clinical Performance and Subjective Outcomes Between Two Diffractive Trifocal Intraocular Lenses (IOLS) and One Monofocal IOL in Bilateral Cataract Surgery. *Frontiers in Bioscience (Landmark Edition)*.

[17] Rosen E., Alio J. L., Dick B. H., Dell S., Slade S. (2016). Efficacy and Safety of Multifocal Intraocular Lenses Following Cataract and Refractive Lens Exchange: Metaanalysis of Peer-Reviewed Publications. *Journal of Cataract & Refractive Surgery*.

[18] Chang J. S. M., Liu S. C. T., Ma N. T. C., Ng J. C. M. (2023). Clinical Outcome of a Quadrifocal (Trifocal) Intraocular Lens in Chinese Patients: Prospective, Observational Case Series. *Journal of Cataract & Refractive Surgery*.

[19] Boris M., Olga F., Nikolay S. (2024). Visual Results and Subjective Satisfaction After Implantation of Two Different Trifocal Diffractive Intraocular Lenses Models (Acrysof IQ Panoptix and AT LISA Tri 839 MP). *European Journal of Ophthalmology*.

[20] Chen X.-Y., Wang Y.-C., Zhao T.-Y., Wang Z.-Z., Wang W. (2022). Tilt and Decentration With Various Intraocular Lenses: A Narrative Review. *World Journal of Clinical Cases*.

[21] Dick H. B., Ang R. E., Corbett D. (2022). Comparison of 3-Month Visual Outcomes of a New Multifocal Intraocular Lens vs a Trifocal Intraocular Lens. *Journal of Cataract & Refractive Surgery*.

[22] Ferreira T. B., Ribeiro F. J., Silva D., Matos A. C., Gaspar S., Almeida S. (2022). Comparison of Refractive and Visual Outcomes of 3 Presbyopia-Correcting Intraocular Lenses. *Journal of Cataract & Refractive Surgery*.

[23] Hida W. T., Moscovici B. K., Cortez C. M. (2024). Comparison of Visual Outcomes of Bilateral Dual-Technology Diffractive Intraocular Lens vs Blended Enhanced Monofocal With Dual-Technology Intraocular Lens. *Journal of Cataract & Refractive Surgery*.

[24] Ribeiro F. J., Ferreira T. B., Silva D., Matos A. C., Gaspar S. (2021). Visual Outcomes and Patient Satisfaction After Implantation of a Presbyopia-Correcting Intraocular Lens That Combines Extended Depth-of-Focus and Multifocal Profiles. *Journal of Cataract & Refractive Surgery*.

[25] Bayhan H. A., Taşcı Y. Y., Aslan Bayhan S., Takmaz T., Can İ. (2024). Comparison of Two Presbyopia-Correcting Trifocal Intraocular Lenses: a Prospective Study. *Turkish Journal of Ophthalmology*.

[26] Kohnen T., Herzog M., Hemkeppler E. (2017). Visual Performance of a Quadrifocal (Trifocal) Intraocular Lens Following Removal of the Crystalline Lens. *American Journal of Ophthalmology*.

[27] Modi S., Lehmann R., Maxwell A. (2021). Visual and Patient-Reported Outcomes of a Diffractive Trifocal Intraocular Lens Compared With Those of a Monofocal Intraocular Lens. *Ophthalmology*.

[28] Schallhorn J. M. (2021). Multifocal and Extended Depth of Focus Intraocular Lenses: A Comparison of Data From the United States Food and Drug Administration Premarket Approval Trials. *Journal of Refractive Surgery*.

[29] Zhong Y., Wang K., Yu X., Liu X., Yao K. (2021). Comparison of Trifocal or Hybrid Multifocal-Extended Depth of Focus Intraocular Lenses: A Systematic Review and Meta-Analysis. *Scientific Reports*.

[30] Asena B. S. (2019). Visual and Refractive Outcomes, Spectacle Independence, and Visual Disturbances After Cataract or Refractive Lens Exchange Surgery: Comparison of 2 Trifocal Intraocular Lenses. *Journal of Cataract & Refractive Surgery*.

